# Two-stage surgery for acute type B aortic dissection and aortic root aneurysm in a patient with Turner syndrome: a case report

**DOI:** 10.1186/s44215-025-00202-9

**Published:** 2025-05-01

**Authors:** Kazunori Sakaguchi, Hidekazu Nakai, Takanori Tsujimoto, Atsunori Kono, Yojiro Koda, Katsuhiro Yamanaka, Kenji Okada

**Affiliations:** Division of Cardiovascular Surgery, Department of Surgery, Kobe University Graduate School of Medicine, 7 - 5- 2 Kusunoki-cho, Kobe, Japan

**Keywords:** Turner syndrome, Type B aortic dissection, Aortic root aneurysm

## Abstract

**Background:**

Patients with Turner syndrome are at high risk of aortic dissection and are more likely to present with fatal outcomes. Turner syndrome is classified as a heritable thoracic aortic disease in the latest EACTS/STS guidelines. Herein, we present a case of two-staged surgery for acute type B aortic dissection and aortic root aneurysm in a patient with Turner syndrome.

**Case presentation:**

A 29-year-old female patient with Turner syndrome was admitted to our center due to back pain and was diagnosed with acute type B aortic dissection. Echocardiography revealed a dilated aortic root with bicuspid aortic valve. On the 5 th day after admission, the patient presented with a higher level of back pain. Follow-up computed tomography scan revealed changes from two- to three-channel aortic dissection a further aortic dilation. Therefore, descending aorta and partial aortic arch replacement were initially performed. Then, valve-sparing aortic root replacement and residual arch replacement were performed 3 months after the first surgery. Postoperative echocardiography confirmed the absence of aortic regurgitation. The patient was discharged on the 17th postoperative day without any complications.

**Conclusions:**

Two-stage surgery was successfully performed for the thoracic aorta and aortic root aneurysms in a patient with Turner syndrome. The patient recovered for 3 months after the left thoracotomy surgery and underwent a two-stage surgery through a median sternotomy surgery with good surgical results.

## Background

Turner syndrome (TS) is a disease caused by an X chromosome abnormality and is characterized by short stature, gender dysgenesis, and cardiovascular disorders. Turner syndrome is classified as a heritable thoracic aortic disease (HTAD) in the latest EACTS/STS guidelines [1]. Vascular complications often occur at a young age, and the mortality of aortic dissection is 58% [2]. Patients with TS often require extensive and multiple surgeries with precise timing for vascular complications. However, only a few reports have investigated the timing of these surgeries in patients with TS. Herein, we present a 29-year-old female patient with TS that was successfully managed with surgeries.

## Case presentation

**Fig. 1:**
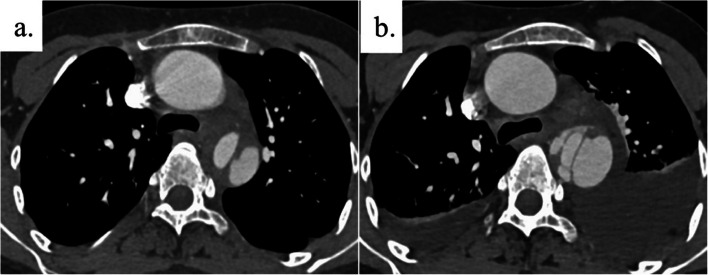
**a** The initial CT scan upon admission showed Stanford type B acute aortic dissection. The diameter of the descending aorta was 36 × 34 mm. **b** Follow-up CT scan on day 5 after the onset revealed a three-channel aortic dissection in the descending aorta, an increased pleural effusion, and a dilated descending aorta (39 × 36 mm)

**Fig. 2:**
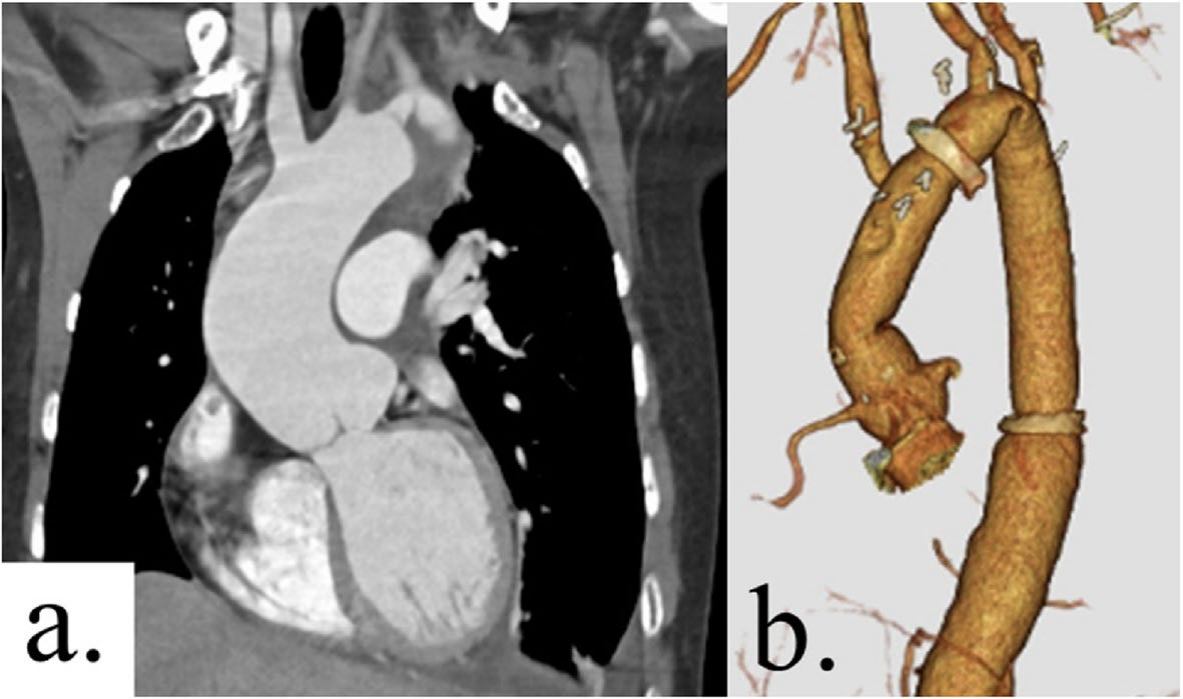
**a** The initial CT scan upon admission showed a dilated aortic root measuring 47 mm. **b** Postoperative three-dimensional CT scan

A 29-year-old female patient with TS was admitted due to back pain. She was diagnosed with Turner syndrome through chromosomal testing at birth. She was 152 cm in height and weighed 54.4 kg. After admission, computed tomography (CT) scan revealed acute type B aortic dissection from the left subclavian artery to the level of the renal artery and aortic root dilatation. At this point, the diameter of the descending aorta was 36 × 34 mm. The patient’s blood pressure was well controlled, and there was no remarkable symptom after admission. However, on the 5 th day after admission, she exhibited a higher level of back pain. Follow-up CT scan showed a three-channel aortic dissection, an increased pleural effusion, and a dilated descending aorta (39 × 36 mm) (Fig. 1). The aortic root size was 47 mm, and the aortic root had a high aortic size index (ASI) at 3.2 cm/m^2^ (Fig. 2a). Moreover, echocardiography revealed that the aortic annulus (22 mm) of bicuspid aortic valve was normal, and there was no evidence of aortic regurgitation (AR). The descending aorta was treated first because it was at a higher risk of malperfusion or rupture compared to the aortic root. Then, the aortic root aneurysm was treated.

Surgery was performed via the posterolateral thoracotomy while the patient was in the right lateral recumbent position. The chest cavity was opened via the 4 th intercostal space with transection of the 4 th and 5 th ribs. Serosanguineous pleural effusion was observed in the left thoracic cavity, but no hemorrhage suggestive of rupture was detected. Cardiopulmonary bypass was established via the femoral artery and vein. A left heart venting tube was inserted through the left atrial appendage to prevent excessive left ventricular distension. Just prior to circulatory arrest, 50 mEq of potassium chloride was administered into the cardiopulmonary circuit to induce diastolic cardiac arrest. The circulation was arrested by moderate hypothermia (rectal temperature at 30 °C), the descending aorta was clamped, and lower extremity perfusion was started. The distal aortic arch was opened to perform selective cerebral perfusion using balloon tipped catheters. The aortic dissection extended up to beside the left subclavian artery ostium. Hence, the open proximal anastomosis between the brachiocephalic artery and the left common carotid artery was performed, and the left common carotid artery and the left subclavian artery were reconstructed. The preoperative CT scan showed that the Adamkiewicz artery originated at the Th10 level. Considering the possibility that reconstructing the intercostal arteries might be challenging due to acute aortic dissection, the extent of the replacement was limited to the proximal side, above the level of the eighth thoracic vertebra. The aorta was replaced up to the level of the eighth thoracic aortic level using the 20-mm Triplex 4-branch graft (Terumo, Tokyo, Japan), with a double-barreled distal anastomosis. The operation time was 547 min, the cardiopulmonary bypass time was 249 min, and the myocardial ischemia time was 78 min. The patient was discharged from the hospital on postoperative day 16 without any complications.

**Fig. 3:**
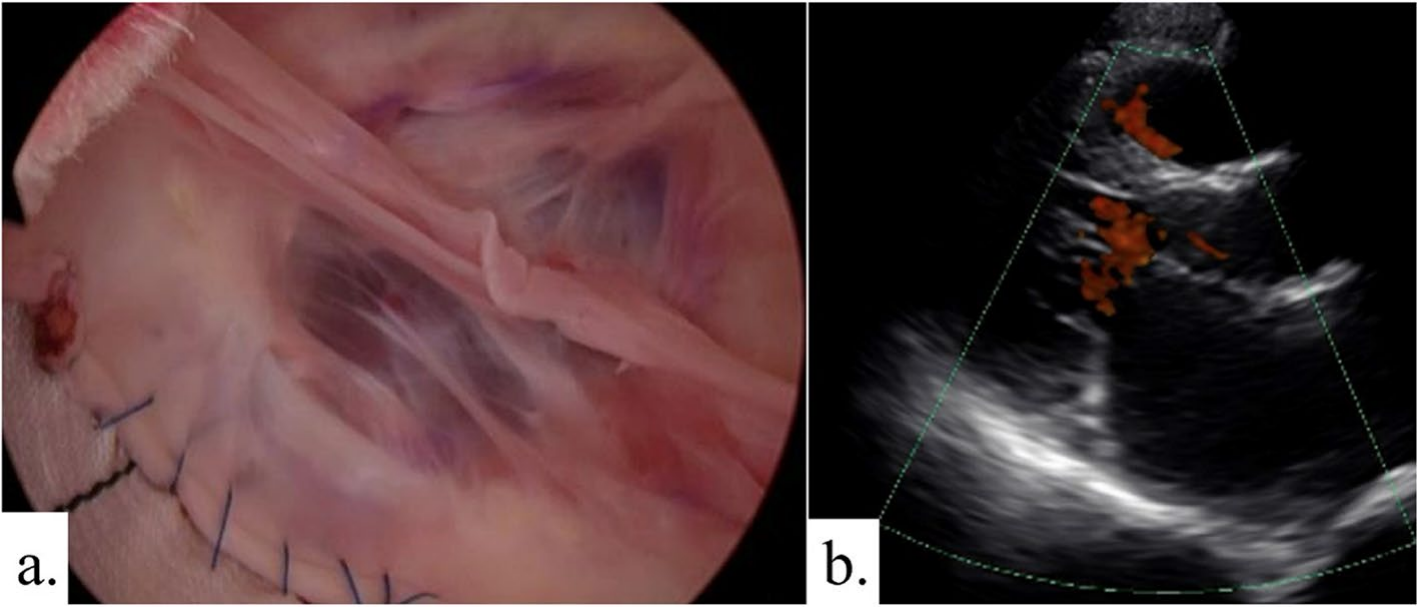
**a** Operative endoscopic view of aortic valve shows no prolapse and good valvular coaptation. **b** Postoperative transthoracic echocardiography showed no evidence of aortic regurgitation

**Fig. 4:**
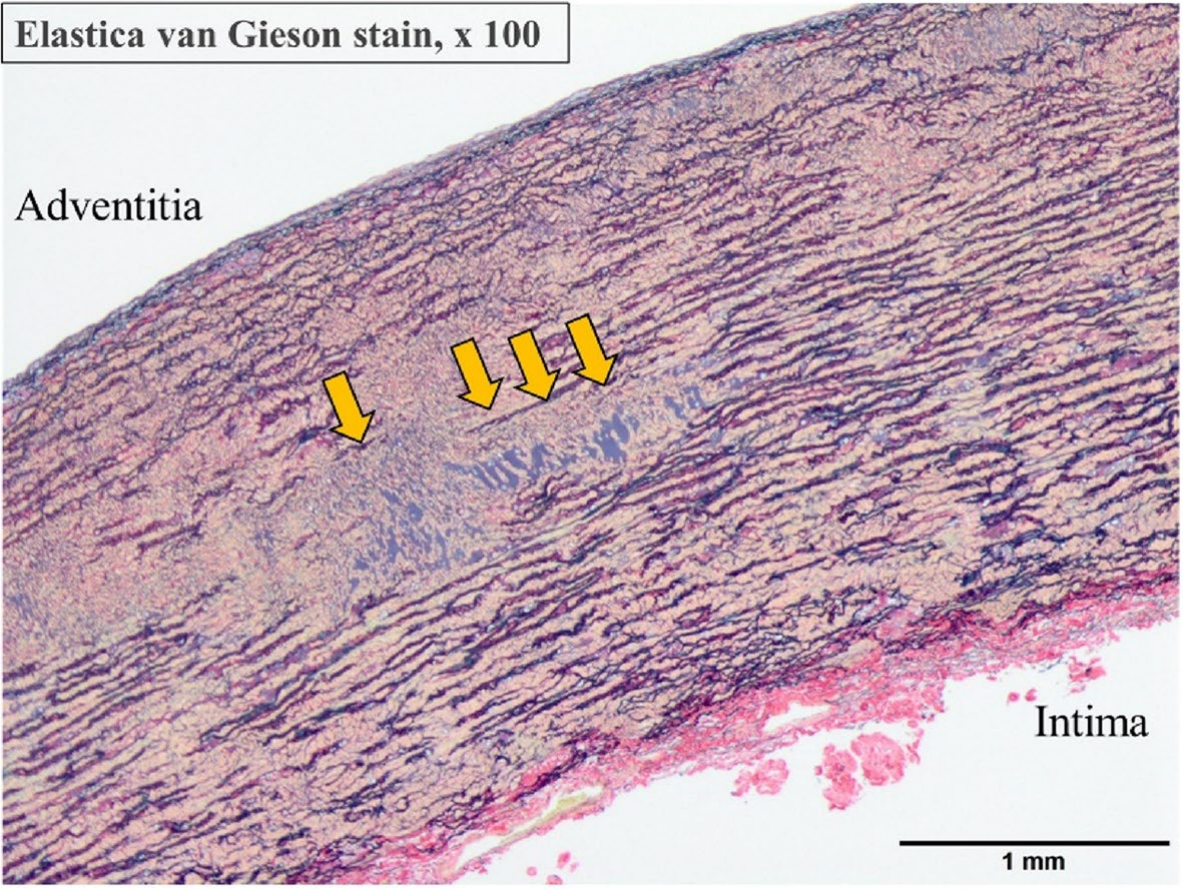
Histological findings of the aortic wall of the ST junction. Elastica van Gieson stain, 100 × magnification. All layers of the aorta are observed. The adventitia shows mild vascular growth and inflammatory infiltration. In the tunica media, elastic fibers are torn and degenerated, and cystic medial necrosis (yellow arrows) can be observed. The intima presents mild fibrotic thickening

After careful monitoring of the patient’s physical and mental condition, a surgical plan was made at 2 months postoperatively, and the second surgery was performed 3 months after the initial surgery. During this period, coronary angiogram also revealed a coronary artery fistula. She underwent surgery for aortic root aneurysm and coronary artery fistula via median sternotomy. Cardiopulmonary bypass was established with cannulation of the ascending aorta and the bicaval drainage. Coronary arteries fistula entering the pulmonary artery was easily identified, and the shunt vessels were ligated. Under ascending aorta clamping, the ascending aorta was transected above the sinotubular junction. A type 1 (R-N) bicuspid aortic valve was observed, and the leaflets were in good condition. Valve-sparing root replacement was performed with the 24-mm Valsalva graft (Terumo, Tokyo, Japan) (Fig. 3a). Under moderate hypothermic circulatory arrest (rectal temperature at 30 °C), the previous anastomosis was opened to perform selective cerebral perfusion using a balloon tipped catheter. Distal anastomosis of the aorta just distal to the brachiocephalic artery was performed with the 22-mm Triplex 4-branch graft (Terumo, Tokyo, Japan). The brachiocephalic artery was reconstructed. The procedural details were as follows: the operation time was 631 min, with 394 min of cardiopulmonary bypass time and 260 min of myocardial ischemia time. The patient was discharged from the hospital on postoperative day 17 without any complications. Postoperative echocardiography did not show evidence of aortic regurgitation (Fig. 3b). Figure 2b shows the postoperative CT angiography images. Figure 4 shows the histological findings of the ascending aortic wall above the ST junction (Elastica van Gieson stain × 100). All layers of the aorta are observed. In the tunica media, elastic fibers are torn and degenerated, and cystic medial necrosis (yellow arrows) can be seen. Three years after the surgery, the patient has been free from aortic events.

## Discussion

Patients with TS have a 100-fold higher risk of aortic dissection than the general female population [2]. Further, they are at a higher risk of fatal complication of aortic dissection, and they exhibit pathological findings similar to those of Marfan syndrome (MFS) [3–5]. Thoracic endovascular aortic repair is generally avoided in patients with MFS due to the high rate of aortic events. Previous studies have reported fatalities in patients with TS undergoing thoracic endovascular aortic repair for type B aortic dissection [6, 7]. The descending aorta and partial arch were replaced with a left open chest to safely perform the next surgery.

Although the proximal circulatory arrest time of 78 min was long, we considered it to be within the acceptable limit as long as the heart was arrested [8]. If the proximal circulatory arrest had been prolonged further, occlusion balloon would have been used to administer cardioplegia.

There was no aortic regurgitation in this case; however, aortic regurgitation is considered a common coexistent condition in many cases of root dilatation. In cases of mild or moderate aortic regurgitation (AR), insertion of a left heart venting tube is planned to avoid overdilation of the left ventricle. If the AR is more severe or if more concern is given to myocardial protection, Anterolateral partial sternotomy is a reliable approach. For instance, it facilitates ascending aortic cross-clamping and the administration of myocardial protective solution, particularly for selective antegrade coronary perfusion or retrograde cardioplegia [9].

TS with aortic dissection is associated with high mortality [3]. Therefore, intervention for the dilated aortic root was carefully considered, and the two-stage surgery was performed to avoid further aortic events. However, there are only a few reports on staged surgery for aortic lesions in TS. Ikeno reported the outcomes of staged surgeries in patients with MFS, with an average interval of 4.8 years between the first and second surgeries. However, among the previous cases, one patient died from rupture of a residual thoracic aortic aneurysm 10 months after the initial surgery. Thus, the optimal interval between surgeries remains unclear [10]. In this case, a two-stage surgery was performed after 3 months, and the surgical outcome was favorable.

TS is characterized by a short stature. Therefore, the same criteria for aortic diameter in Marfan syndrome cannot be applied. The 2014 ESC guidelines presented the indications for surgery, considering indexed ASI according to BSA [2, 11]. We used the indexed aortic diameter to identify surgical interventions for the aortic root when discussing surgery in this case. However, the newest EACTS/STS guidelines now focus on ascending aortic length as a risk factor for aortic aneurysm [12]. Therefore, noteworthily, the guidelines indicate that the aortic diameter should be corrected for height alone, to obviate weight oscillations. Additionally, the guidelines introduce the term heritable thoracic aortic disease and list MFS, Loeys–Dietz syndrome, vascular Ehlers–Danlos syndrome, and TS. Although no individual recommendation is given for TS, it can be considered an indication for surgery as HTAD. Among MFS, a family history of aortic dissection, annual enlargement of > 0.3 cm, diffuse aortic root, and ascending aortic dilation are considered high-risk features. Surgical intervention may be considered for patients with MFS who have these risks when the aortic root or ascending aorta is ≥ 45 mm (class 2a). This patient has already had a type B aortic dissection; thus, she is at high risk. Therefore, the latest guidelines recommend aortic root surgery. Thus, specific aortic diameters for surgical intervention in HTAD are now being developed for each syndrome. We hope to accumulate more results in the future.

The 2022 ACC/AHA guideline recommends that patients with Turner syndrome who are aged ≥ 15 years and have an aortic size index ASI of ≥ 2.5 cm/m^2^, along with risk factors for aortic dissection, should undergo surgical intervention to replace the aortic root, ascending aorta, or both (class 2a recommendation) [13]. This patient is 29 years old with an ASI of 3.2 cm/m^2^ at the aortic root. Therefore, this guideline recommends surgery.

Valve-sparing root replacement (VSRR) is the standard surgery for annuloaortic ectasia and is effective in patients with connective tissue disease [14, 15]. The aortic valve was a type 1 bicuspid valve. However, VSRR could be performed because there were no signs of valve cusp degeneration and aortic valve regurgitation.

The prevalence of risk factors associated with aortic aneurysms and dissections, including bicuspid aortic valve, hypertension, and coarctation of the aorta, is high in patients with TS [16]. Further, TS itself is an independent risk factor of aortic dilation [17]. In this case, the presence of a bicuspid aortic valve indicated a higher risk of aortic events. Thus, close observation with imaging assessments at an outpatient clinic is required.

## Conclusions

We present the successful two-staged surgery for acute type B aortic dissection and aortic root aneurysm in a patient with Turner syndrome.

## Data Availability

Not applicable.

## Abbreviations

AR: Aortic regurgitation
ASI: Aortic size index
CT: Computed tomography
HTAD: Heritable thoracic aortic disease
MFS: Marfan syndrome
TS: Turner syndrome
VSRR: Valve-sparing root replacement
